# CMKLR1 deficiency maintains ovarian steroid production in mice treated chronically with dihydrotestosterone

**DOI:** 10.1038/srep21328

**Published:** 2016-02-19

**Authors:** Mi Tang, Chen Huang, Yu-Fei Wang, Pei-Gen Ren, Li Chen, Tian-Xia Xiao, Bao-Bei Wang, Yan-Fei Pan, Benjamin K. Tsang, Brian A Zabel, Bao-Hua Ma, Hui-Ying Zhao, Jian V. Zhang

**Affiliations:** 1 College of Veterinary Medicine, Northwest Sci-Tech University of A&F, Yangling, Shanxi, 712100, China; 2 Research Laboratory for Reproductive Health, Shenzhen Institute of Advanced Technology, Chinese Academy of Sciences, Shenzhen 518055, China; 3 University of Science and Technology of China, An-Hui, He-Fei, 230026, China; 4 Palo Alto Veterans Institute for Research, Veterans Affairs Palo Alto Health Care System, Palo Alto, CA, USA; 5 Department of Obstetrics & Gynaecology, University of Ottawa, Ottawa, Ontario K1H 8L6, Canada; 6 Chronic Disease Program, Ottawa Hospital Research Institute Ontario K1H 8L6, Canada; 7 Department of Cellular & Molecular Medicine, University of Ottawa; Ottawa, Ontario K1H 8L6, Canada; 8 Interdisciplinary School of Health Sciences, University of Ottawa, Ottawa, Ontario K1H 8L6, Canada

## Abstract

Elevated serum chemerin levels correlate with increased severity of polycystic ovary syndrome (PCOS). However, the role of CMKLR1 signaling in ovarian biology under conditions of excess DHT remains unclear. In this study we compared the effects of continuous 90-day high dose DHT exposure (83.3 □g/day) on wild type and CMKLR1-deficient mice. DHT induced PCOS-like clinical signs in wild type mice as well as significant changes in the expression of hormone receptors, steroid synthesis enzymes, and BMPs and their receptors. In contrast, CMKLR1-deficient mice significantly attenuated DHT-induced clinical signs of PCOS and alterations in ovarian gene expression. To determine whether the BMP4 signaling pathway was involved in the pathogenic effects of CMKLR1 signaling in DHT-induced ovarian steroidogenesis, antral follicles were isolated from wild type and CMKLR1 knockout (KO) mice and treated *in vitro* with combinations of hCG, DHT, and BMP4 inhibitors. BMP4 inhibition attenuated the induction effects of hCG and DHT on estrogen and progesterone secretion in CMKLR1 KO mice, but not in WT mice, implicating the BMP4 signaling pathway in the CMKLR1-dependent response to DHT. In conclusion, CMKLR1 gene deletion attenuates the effects of chronic DHT treatment on ovarian function in experimental PCOS, likely via BMP4 signaling.

In mammals, regular estrous cycles depend on highly regulated production of estrogen and progesterone, and the synthesis and secretion of estrogen and progesterone are dependent on the normal development of follicles and ovulation. Exposure to high concentrations of androgens can cause abnormal follicular development and estrogen and progesterone secretion disorders, leading to abnormal estrous cycles and constituting the symptoms of polycystic ovary syndrome (PCOS).

Chemokine-like receptor-1 (CMKLR1), an orphan G-protein-coupled receptor (GPCR), is specifically expressed by monocyte-derived dendritic cells, macrophages, and circulating plasmacytoid dendritic cells (pDCs)^1^,^2^. Several groups have reported that CMKLR1 mRNA is also expressed in adipose tissue, liver, and the testes and ovaries of humans, rats, and mice^1^,^3^,^4^,^5^. The natural ligand for CMKLR1, chemerin, was recently discovered^1^,^3^,^6^. Chemerin has been isolated from ascitic fluid (ovarian carcinoma), inflamed synovial fluid, hemofiltrate, and normal serum. Chemerin was identified as a chemoattractant ligand for CMKLR1^3^,^6^ but has also been found to be a novel adipokine associated with obesity and metabolic syndrome and to promote adipogenesis and regulate glucose metabolism^3^,^4^,^7^,^8^.

Serum chemerin levels are higher in obese women and in women with PCOS^4^,^9^, and metformin (a drug for the treatment of PCOS patients with insulin resistance) decreases serum chemerin levels^9^, implying a correlation between chemerin and PCOS^10^,^11^. In a rat model of PCOS, in which exposure to 5α-dihydrotestosterone (DHT)^12^ recapitulates the reproductive and metabolic phenotypes of human PCOS^12^, DHT treatment resulted in increased expression of chemerin and CMKLR1 in antral follicles^13^. Moreover, recombinant chemerin was found to suppress both basal estradiol secretion in granulosa cells from normal rats and FSH-induced progesterone and estradiol secretion in cultured preantral follicles and granulosa cells *in vitro*, suggesting the possible involvement of chemerin in the regulation of ovarian follicle growth and function. However, the role of chemerin and its receptor, CMKLR1, in follicular development and the pathogenesis of PCOS remains unclear^13^,^14^. BMP members are co-expressed in the ovary, and thecal interstitial cells are known to produce at least BMP4, BMP2, BMP7, and GDF10^15^,^16^,^17^ It has been reported that BMP4 inhibits progesterone synthesis and secretion in ovine ovarian and bovine granulosa cells but that it has no effect on estradiol^18^.

Here, we hypothesize that androgen-induced ovarian follicular growth and function depends, in part, on increased expression and action of chemerin and the BMP4, ActR IIA, and Alk6 signaling pathways. In this study we used CMKLR1 knockout (KO) mice to investigate the role of the receptor in the DHT induction model of PCOS that mimics the reproductive and metabolic characteristics associated with the human disease^19^,^20^. We also used freshly isolated ovarian antrals from wild type and CMKLR1 KO mice to explore the intersecting molecular signaling pathways activated by hyperandrogenic stimulation.

## Materials and Methods

### Animals

CMKLR1 knockout mice in the C57BL/6J background were provided by Deltagen Ltd. and the Zabel lab^21^. C57BL/6J wild type female mice were obtained from the Laboratory Animal Center, Institutes of Biomedicine and Health, Chinese Academy of Sciences, China. The animals were housed at constant temperature and humidity under a 12-hour light-dark cycle. Chow and water were available *ad libitum*. All animal procedures were carried out in accordance with the approved guildellines by the Committee on the Use of Live Animals for Teaching and Research, Shenzhen Institutes of Advanced Technology, Chinese Academy of Sciences. At postnatal d 19, mice of comparable body weights were randomly divided into two treatment groups (DHT and control; 10 per group) and were implanted *s.c.* with a 90-d DHT continuous-release pellet (Innovative Research of America, Sarasota, FL). These pellets contained 7.5 mg of DHT (daily dose, 83.3 μg). Control mice received a placebo pellet. Mice were sacrificed at the end of the treatment period (90 d). Body weight was determined at the start and end of treatment. In addition, at the end of the 90-d treatment period, blood samples and tissues were collected. Blood samples were collected by orbital puncture after the mice were anesthetized with isoflurane. Ovaries and uteri were isolated, weighed, and fixed overnight in Bouin’s fluid. Gonadal fat depots were isolated and fixed overnight in 4% paraformaldehyde. In addition, isolated tissues were snap frozen in liquid nitrogen and stored at −80 °C until further processing.

### Estrous Cycle determination

To determine stage in the estrous cycle, daily vaginal smears were taken 12 d before the animals were killed and examined.

### Ovarian histology

For histological examination of ovarian morphology, fixed ovaries were embedded in paraffin. After routine histological procedures, 5 μm sections were mounted on glass slides and stained with hematoxylin and eosin. Follicle counting was performed in serial ovarian sections. In brief, based on the mean diameters of the follicles, growing follicles were divided into four classes: small preantral (20–170 μm), large preantral (171–220 μm), small antral (221–310 μm), and large antral (311 μm). Non-atretic and atretic growing follicles were counted in every fifth section. Primordial follicles were counted in every second section. In addition, sections were examined for the presence of recent corpora lutea.

### TUNEL assay

Five μm ovarian sections were mounted on glass slides, and the DNA Fragmentation Detection Kit (QIA 39, Calbiochem, Germany) was used to quantify DNA ends generated in response to apoptotic signals.

### Real-time PCR

Total RNA from tissues and cells was extracted using RNA iso Plus reagent and subjected to real-time RT-PCR analysis. RNA samples (1 μg) were reverse transcribed into cDNA according to the manufacturer’s instructions (Bio-Rad Laboratories, Hercules, CA). The PCR reaction mixtures contained 10 μl SYBR® Premix Ex TaqTM II (Takara, Japan), 500 nM of each primer, 1 μl template cDNA, and DNase-free water to a final volume of 20 μl. Cycle conditions were 95 °C for 10 sec, followed by 45 cycles of 95 °C for 5 sec, 60 °C for 30 sec, and 72 °C for 30 sec. The reaction was completed with a dissociation step for melting point analysis from 50 °C to 95 °C (in increments of 0.5 °C for 10 sec each). The primer sequences and their reference sequences are presented in Table 1. Gene expression levels were normalized to levels of β-actin using the ΔCT method, where CT was the cycle threshold. Melting curve analysis for each primer set revealed only one peak for each product. The size of the PCR products was confirmed by comparing the size of product with a commercial ladder after agarose gel electrophoresis

### ELISA and RIA

DHT levels were measured with a DHT ELISA kit (Diagnostics Biochem Canada, Inc., London, Ontario, Canada). Progesterone, estradiol, LH, and FSH levels in sera were measured using commercial Iodine [^125^I] Radioimmunoassay Kits (Lareneen, GZ, China). The intra- and inter-assay errors among all assays were less than 10% and 15%, respectively.

### Follicle culture

Superovulation was induced in 19-day-old immature female C57BL/6 J mice by intraperitoneal (i.p.) injection with 5 IU per mouse of pregnant mare serum gonadotropin (Sigma, PMSG). Large antral follicles of > 300 μm diameter were isolated 48 h post-PMSG treatment. Ovarian follicles were isolated in ice-cold normal saline with a dissecting microscope and two 26 gauge 1/2 inch syringe needles and pre-incubated at 37 °C in 24-well plates (3 follicles per well) containing DMEM/F12-0.1% BSA. The follicles were then treated with 0.01 IU/ml hCG or 1 μg/mL DAN (D2066, Sigma) in the presence of 1 μM DHT for 24 h. Conditioned media were collected for progesterone and estradiol measurement. Follicles were lysed for real-time PCR analysis.

### Statistics

Data were statistically analyzed by Duncan’s multiple range test for individual comparison repeated measures ANOVA, or two-way ANOVA (adipocyte size distribution), using SPSS 15.0 (SPSS, Inc., Chicago, IL). Data are expressed as means ± SEM, and differences were considered significant at *p* < 0.05.

## Results

### Cyclicity, ovarian morphology, and serum hormones

**Figure 1:**
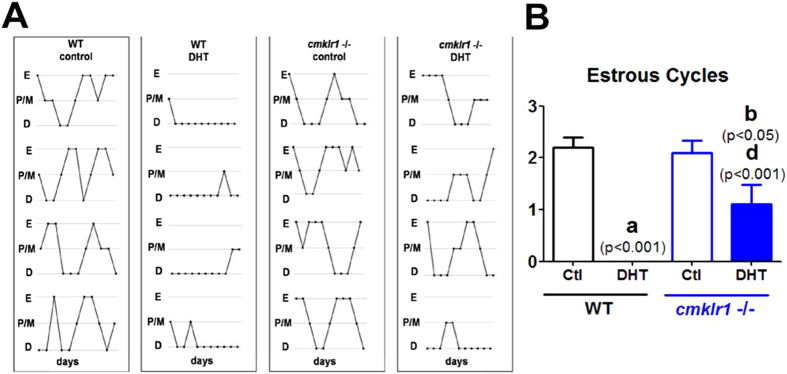
Estrous cycles were monitored for 12 days in CTL (placebo) and DHT-treated wild type and CMKLR1 null mice (n = 10). (**A**) Both control wild type and CMKLR1 null mice underwent 2–3 regular estrous cycles. Estrous cycles were absent in DHT-treated wild type mice; however, DHT-treated CMKLR1 null mice underwent 1–2 estrous cycles. (**B**) Quantification showed that CMKLR1 deficiency prevented the acyclicity induced by DHT. E, estrous cycle; P/M, proestrous cycle and metaestrous cycle; D, diestrous cycle. WT control, placebo-treated wild type mice; WT-DHT, DHT-treated wild type mice; cmklr1−/− control, placebo-treated CMKLR1 null mice; cmklr1−/− DHT, DHT-treated CMKLR1 null mice. Data were analyzed using an unpaired Student’s t test. (a) *p*, DHT-treated *vs.* control wild type mice; (b) *p*, DHT-treated *vs.* control CMKLR1-null mice; (d) *p*, DHT-treated CMKLR1 null mice *vs.* DHT-treated wild type mice.

**Figure 2:**
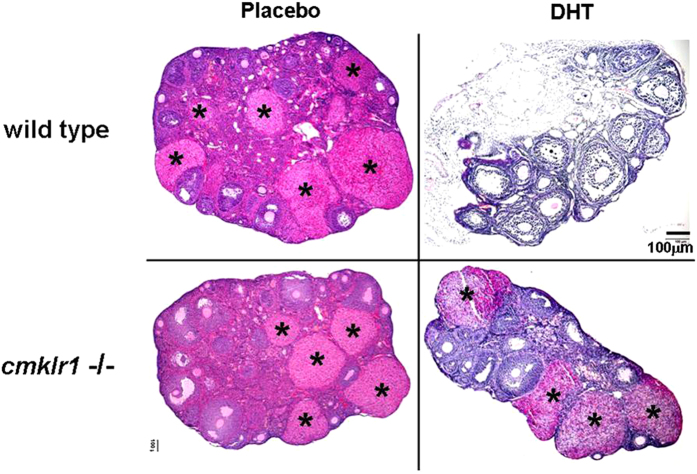
Ovarian features in CTL (placebo) and DHT-treated wild type and CMKLR1-deficient mice. Hematoxylin and eosin staining of CTL and DHT-treated ovaries were observed. Both CTL wild type mice and CMKLR1 null mice display follicles at different stages and the presence of corpora lutea. DHT treatment results in shrinkage of the ovarian structure with minimal large antral follicles and the absence of corpora lutea in wild type mice, whereas DHT-treated CMKLR1-null mice contain a few follicles at different stages and corpora lutea. DHT-treated wild type ovaries contain condensed atypical follicles exhibiting absence of oocytes and reduced granulosa cell numbers. However, DHT-treated CMKLR1 null ovaries display more typical follicles and fewer atypical follicles. The symbol “*” represents CL: corpus luteum. Bar = 100 μm.

**Figure 3:**
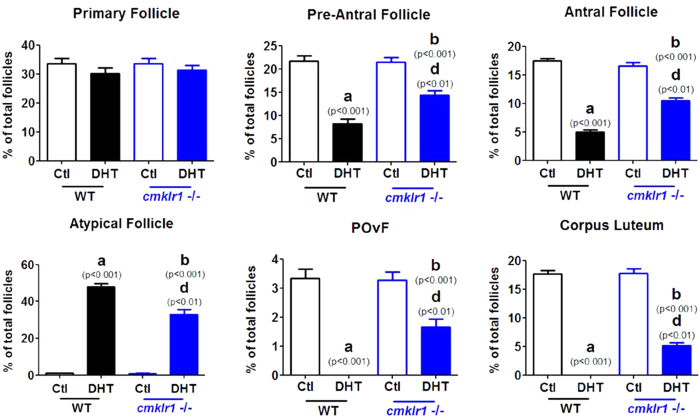
Follicle distribution in CTL (placebo) and DHT-treated wild type and CMKLR1 null mice. Ovaries of DHT-treated wild type mice harbor fewer follicles in preantral to preovulatory stages but a markedly greater number of condensed atypical follicles in comparison to CTLs; these ovaries also lacked corpora lutea. However, ovaries of DHT-treated CMKLR1 null mice contained more preantral, antral, and preovulatory follicles and fewer atypical follicles than ovaries of DHT-treated wild type mice. Data were analyzed by an unpaired Student’s t test. (a) *p*, DHT-treated *vs.* control wild type mice; (b) *p*, DHT-treated *vs.* control CMKLR1 null mice; (d) *p*, DHT-treated CMKLR1 null mice *vs.* DHT-treated wild type mice. POvF = preovulatory follicle.

Like placebo-treated wild type mice, CMKLR1 null mice had regular cycles of 3–5 days and exhibited 2–3 regular estrous cycles during the 12-day observation periods (Fig. 1A). The ovaries of the CMKLR1 null mice contained follicles at different stages of follicular development, as well as fresh corpora lutea, indicative of recent ovulations (Fig. 2). Vaginal smears from WT DHT-treated mice revealed that all mice were in continuous anestrous (Fig. 1A; *p* < 0.001), suggesting that DHT-treated mice were acyclic (Fig. 1A). Consistent with the disruption of the estrous cycle, DHT treatment of wild type mice resulted in shrinkage of the ovarian structure, with minimal large antral follicles and an absence of corpora lutea (Fig. 2). However, DHT-treated CMKLR1 null mice had 1–2 estrous cycles during the 12-day investigation period (Fig. 1A), and their ovaries contained fresh corpora lutea (Fig. 2). DHT-treated wild type mouse ovaries contained fewer follicles in preantral-to-preovulatory stages but numerous condensed atypical follicles and corpora lutea (Fig. 3). In contrast, ovaries from DHT-treated CMKLR1 null mice contained significantly more preantral, antral, and preovulatory follicles and fewer atypical follicles than ovaries from DHT-treated wild type mice (Fig. 3; p < 0.05).

**Figure 4:**
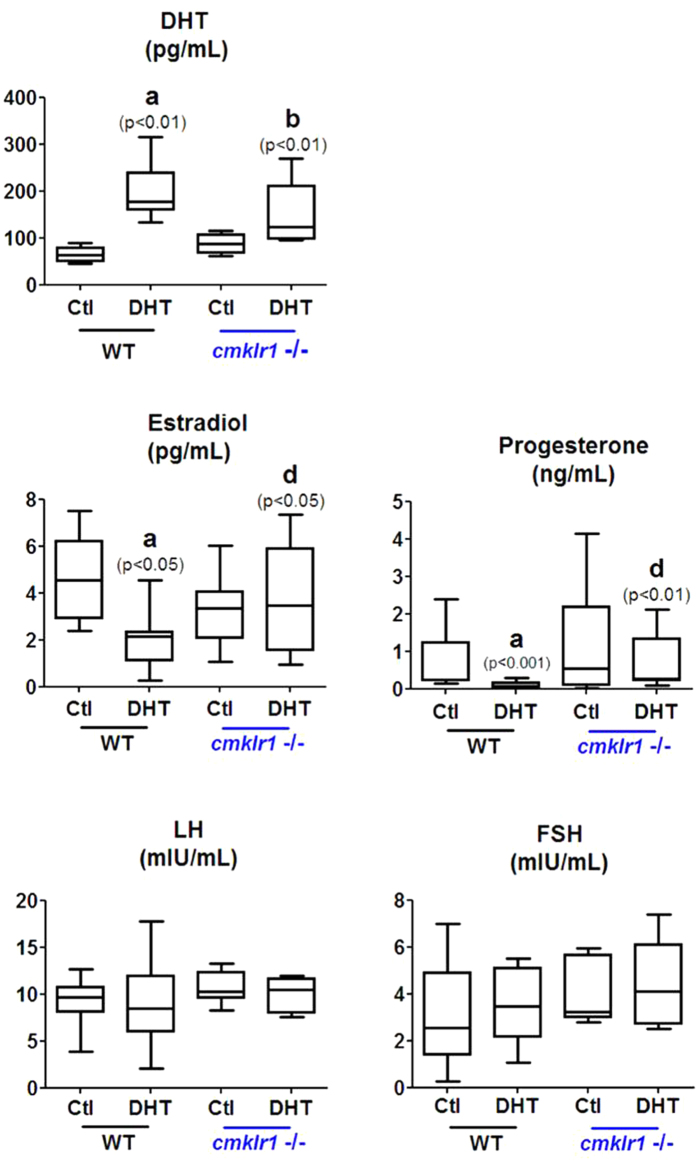
Serum DHT, progesterone, estradiol, LH, and FSH in CTL (placebo) and DHT-treated wild type and CMKLR1-null mice (n = 10). DHT-treated CMKR1 null mice contain lower estradiol and progesterone levels than DHT-treated wild type mice. There was no significant change in serum LH, FSH, or LH/FSH levels in the four groups of mice. Data were analyzed by an unpaired Student’s t test. (a) *p*, DHT-treated *vs.* control wild type mice; (b) *p*, DHT-treated *vs.* control CMKLR1-null mice; (d) *p*, DHT-treated CMKLR1 null mice *vs.* DHT-treated wild type mice.

Next, we measured serum hormone levels in the four groups of mice. DHT levels were significantly elevated in both wild type and CMKLR1 KO mice implanted with DHT-releasing pellets compared with placebo controls, confirming proper establishment of the *in vivo* hyperandrogenism model (Fig. 4). DHT treatment in wild type mice led to lower serum estradiol and progesterone levels than placebo treatment (Fig. 4; p < 0.05 and p < 0.001, respectively). In contrast, DHT-treated CMKLR1 KO mice and placebo CMKLR1 KO controls had similar serum levels of estradiol and progesterone (p > 0.05), and their levels were significantly higher than those of DHT-treated wild type mice (Fig. 4; p < 0.05 and p < 0.01). Serum FSH and LH levels, however, did not differ significantly among the four groups (Fig. 4).

**Figure 5:**
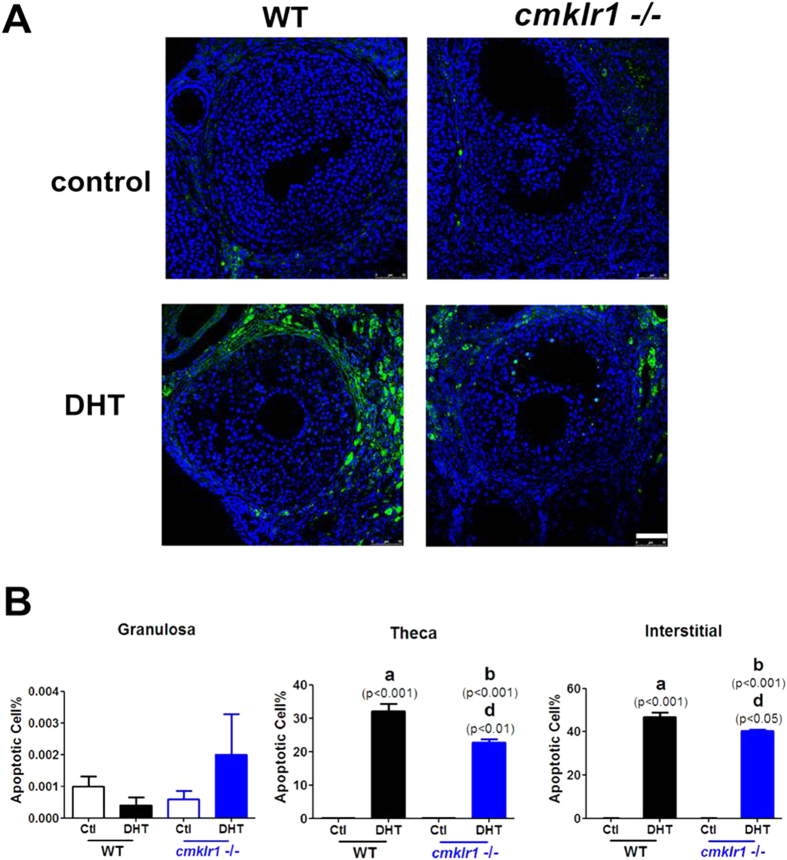
Apoptotic cells in DHT-treated wild type and CMKLR1-deficient mouse ovaries. (**A**) Ovarian tissues of CTL and DHT-treated wild type mice and CMKLR1 null mice labeled by fluorescent TUNEL (green) and DAPI (blue) in interstitial sections of antral follicles. DHT treatment induced dramatically more apoptosis in thecal and interstitial cells of wild type than CMKLR1 null mice. Apoptosis was nearly undetectable in untreated ovaries. (**B**) Data were analyzed by an unpaired Student’s t test. (a) *p*, DHT-treated *vs.* control wild type mice; (b) *p*, DHT-treated *vs.* control CMKLR1 null mice; (d) *p*, DHT-treated CMKLR1 null mice *vs.* DHT-treated wild type mice.

Apoptosis was quantified in antral follicles in adjacent sections in the four experimental groups by terminal deoxynucleotide transferase-mediated dUTP nick end labeling [TUNEL]. (Fig. 5). Compared to follicles in the placebo-treated wild type mouse group, follicles from wild type mice treated with DHT exhibited markedly more apoptotic cells in the thecal layer and interstitial area (Fig. 5A,B; p < 0.001 and p < 0.001, respectively). Furthermore, the follicles of DHT-treated CMKLR1 KO mice had more apoptotic cells in these regions than did those in the placebo-treated CMKLR1 KO mice (Fig. 5A,B; p < 0.001 and p < 0.001, respectively) but significantly fewer than those in DHT-treated wild type mice (Fig. 5A,B; p < 0.01 and p < 0.05, respectively).

### Expression of mRNAs for steroid receptors, steroid synthesis enzymes, and BMPs and their receptors in ovaries

**Figure 6:**
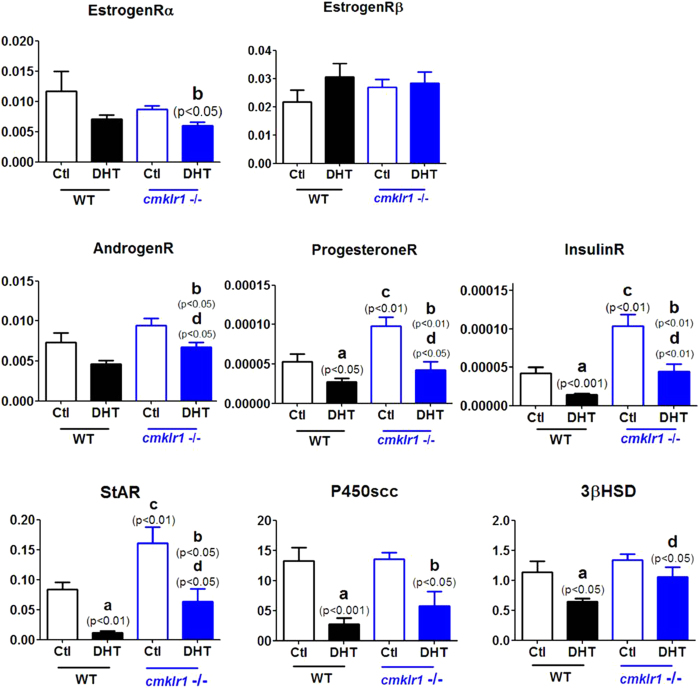
Expression of mRNA for steroid receptors and steroid synthesis enzymes in ovaries of DHT-treated wild type and CMKLR1-deficient mice. Data were analyzed by an unpaired Student’s t test. (a) *p*, DHT-treated *vs.* control wild type mice; (b) *p*, DHT-treated *vs.* control CMKLR1 null mice; (c) *p*, vehicle-treated CMKLR1 null mice *vs.* vehicle-treated wild type mice; (d) *p*, DHT-treated CMKLR1 null mice *vs.* DHT-treated wild type mice.

In order to understand the molecular mechanism underlying the above phenotypes, we further investigated mRNA expression for steroid receptors, steroid synthesis enzymes, and BMPs and their receptors in the ovaries of the four experimental groups (Fig. 6).

Our data revealed no difference between placebo-treated wild type and CMKLR1 KO mice in the abundance of mRNAs for ovarian estrogen receptor-α, estrogen receptor-β, and androgen receptor (Fig. 6). Although DHT had no significant influence on these mRNA levels in wild type mice, it significantly decreased mRNA abundance for ovarian estrogen receptor-α and androgen receptor, although not for estrogen receptor-β, in CMKLR1 KO mice (Fig. 6; p < 0.05 and p < 0.05, respectively). Whereas DHT treatment resulted in a statistically non-significant 40% decrease in ovarian androgen receptor mRNA abundance in wild type mice, the response in the KO mice to DHT was both greater and statistically significant (Fig. 6; p < 0.05).

DHT treatment also led to decreased levels of mRNAs for progesterone receptor, insulin receptor, StAR, and P450scc in the ovaries of both wild type and CMKLR1 KO mice (Fig. 6; p < 0.05, p < 0.001, p < 0.01, and p < 0.001, respectively). Interestingly, DHT administration suppressed the expression of 3βHSD mRNA in the ovaries of wild type mice (Fig. 6; p < 0.05) but not in the ovaries of CMKLR1 null mice. Compared with placebo-treated wild type mice, placebo-treated CMKLR1 null mice had higher ovarian levels of mRNA for progesterone receptor, insulin receptor, and StAR (Fig. 6; p < 0.001, p < 0.01, and p < 0.01, respectively). Moreover, CMKLR1 KO markedly attenuated the DHT-induced downregulation of mRNAs for progesterone receptor, insulin receptor, and StAR (Fig. 6; p < 0.05, p < 0.01, and p < 0.05, respectively). However, CMKLR1 KO did not signifcantly affect the expression of either P450scc or 3βHSD.

**Figure 7:**
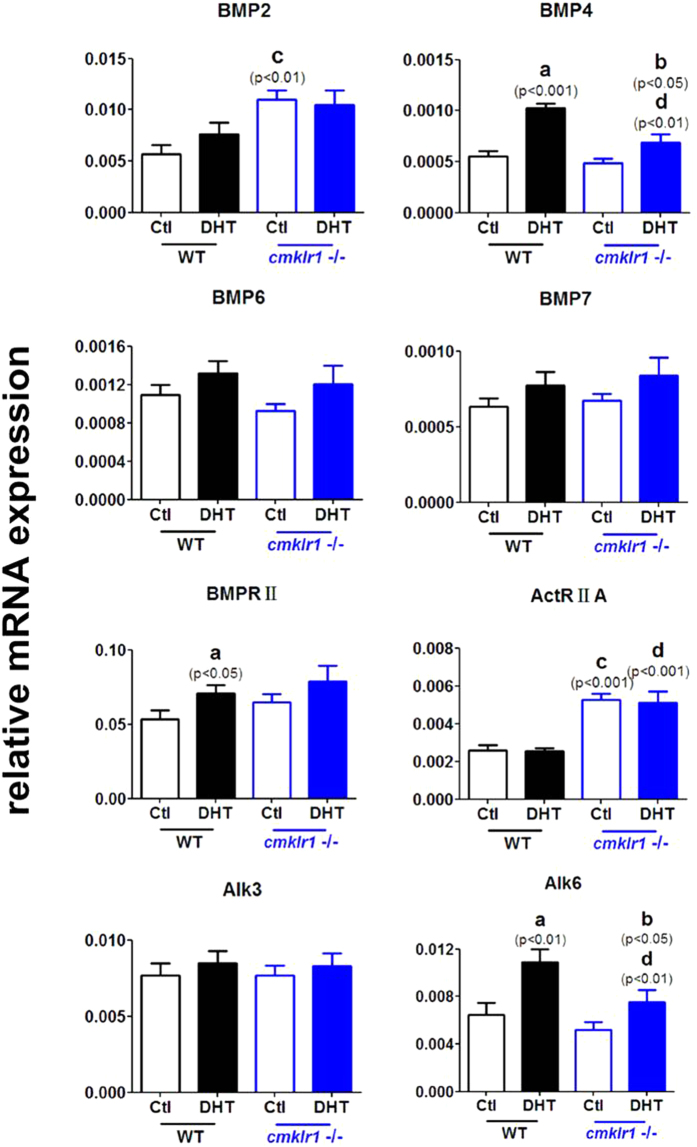
Expression of mRNA for BMPs and their receptors in ovaries of DHT-treated wild type mice and CMKLR1-deficient mice. Data were analyzed by an unpaired Student’s t test. (a) *p*, DHT-treated *vs.* control wild type mice; (b) *p*, DHT-treated *vs.* control CMKLR1 null mice; (c) *p*, vehicle-treated CMKLR1 null mice *vs.* vehicle-treated wild type mice; (d) *p*, DHT-treated CMKLR1 null mice *vs.* DHT-treated wild type mice.

We then investigated BMPs (BMP2, BMP4, BMP6, and BMP7) and their receptors (type II receptor, BMPRII/ActRIIA and type I receptor, Alk3/6) in the ovaries of the four groups (Fig. 7). We found that BMP2 and ActRIIA mRNA levels were higher in the ovaries of placebo-treated CMKLR1 KO mice than in those of placebo-treated wild type mice (Fig. 7; p < 0.01 and p < 0.001, respectively). DHT treatment significantly increased BMP4 and Alk6 expression in the ovaries of both wild type and CMKLR1 KO mice (Fig. 7), although these responses were significantly smaller in the wild type mice (Fig. 7; p < 0.01 and p < 0.01, respectively). There was no significant difference in BMP6, BMP7, and Alk3 mRNA abundance among the four experimental groups. DHT treatment increased BMPRII expression in the ovaries of wild type mice (Fig. 7; p < 0.05) but not in the ovaries of CMKLR1 null mice. ActRIIA expression was higher in the ovaries of DHT-treated CMKLR1 null mice than in those of DHT-treated wild type mice (Fig. 7; p < 0.001).

### Role of BMP4 in DHT-induced, chemerin-mediated ovarian antral follicular steroidogenesis *in vitro*

**Figure 8:**
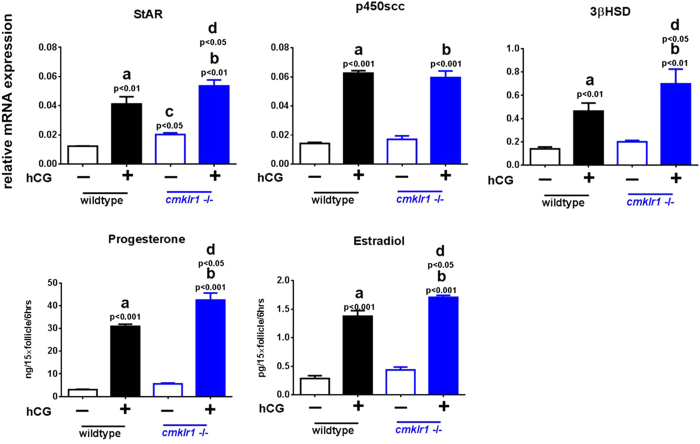
StAR, p450scc, and 3βHSD mRNA expression and estradiol and progesterone production in antral follicles of wild type and CMKLR1 null mice. Follicles were collected after PMSG treatment for 48 h and further treated with 0.01 IU hCG for 6 h. Data were analyzed by an unpaired Student’s t test. (a) *p*, vehicle *vs.* hCG treatment in the wild type follicle group; (b) *p*, vehicle *vs.* hCG treatment in the CMKLR1 null mice follicle group; (c) *p* WT *vs.* CMKLR1 null mice follicles treated with vehicle; (d) *p*, WT *vs.* CMKLR1 null mice follicles treated with hCG.

**Figure 9:**
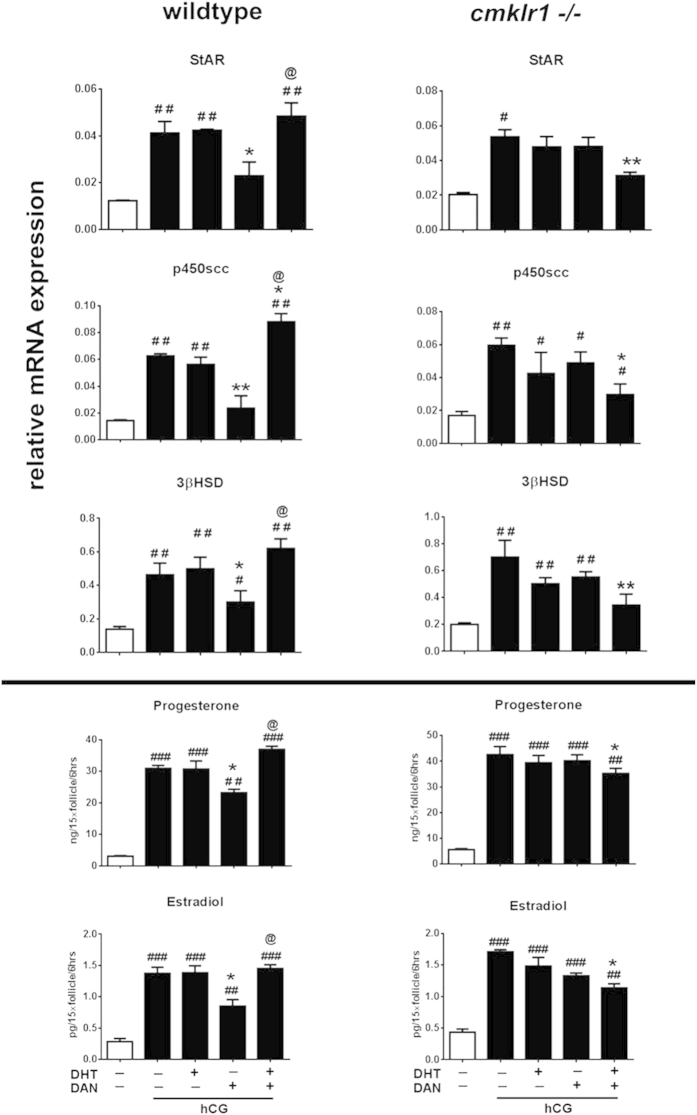
Antral follicles were collected after PMSG treatment for 48 h and further treated under different conditions for 6 h. StAR, p450scc, and 3βHSD mRNA expression and estradiol and progesterone production in antral follicles of wild type mice or CMKLR1 null mice were measured. Data were analyzed by an unpaired Student’s t test. ^#^*p*, compared with non-hCG treatment group (^#^p < 0.05; ^##^p < 0.01; ^##^p < 0.001); *p compared with hCG-treated follicles (*p < 0.05; **p < 0.01; ***p < 0.001); ^@^*p*, compared with DAN-treated follicles in the presence of hCG (^@^p < 0.05).

Since BMP4 and its receptors ActRIIA and Alk6 are expressed differently in DHT-treated CMKLR 1 null mice compared with DHT-treated wild type mice, we further investigated whether BMP4 plays a role in DHT-induced, chemerin-mediated downregulation of antral follicular steroidogenesis. Specifically, we examined the influence of the BMP4 antagonist DAN on the abundance of mRNAs for steroidogenic enzymes and progesterone/estradiol production in antral follicles from PMSG-treated mice cultured with hCG (0.01IU, 6 h) and/or DHT (Figs 8 and 9). As shown in Fig. 8, hCG markedly increased StAR (p < 0.01), p450scc (p < 0.001), and 3βHSD (p < 0.01) mRNA abundance and estradiol (p < 0.001) and progesterone (p < 0.001) secretion in antral follicles from both CMKLR1 wild type and KO mice. With the exception of p450scc, the extent of these responses to hCG appeared greater in CMKLR1 KO mice (Fig. 8).

We then carried out hCG co-treatment with DHT or DAN or with a combination of DHT and DAN in antral follicles (Fig. 9). DHT alone had no effect on the hCG-induced steroidogenic responses in either CMKLR1 or wild type follicles. DAN decreased StAR (p < 0.05), p450scc (p < 0.01), and 3βHSD (p < 0.05) mRNA abundance and inhibited progesterone (p < 0.05) and estradiol (p < 0.05) secretion induced by hCG in wild type but not in CMKLR1 KO follicles. Furthermore, DHT co-treatment reversed the inhibition effect of DAN on hCG-induced steroidogenic responses in wild type follicles, However, the induction effect by hCG was significantly attenuated by co-treatment with DHT and DAN in CMKLR1 KO follicles (Fig. 9; p < 0.05).

Interestingly, treatment of antral follicles from CMKLR1 KO mice with DHT or DAN *in vitro* had no significant influence on hCG induction of either mRNAs for steroidogenic enzymes or progesterone and estradiol secretion. However, together with DHT, DAN markedly inhibited hCG induction of StAR (p < 0.01), p450scc (p < 0.05), and 3βHSD (p < 0.01) mRNA and secretion of progesterone (p < 0.05) and estradiol (p < 0.05).

### Gonadal WAT morphology and expression of mRNA for steroid receptors and WAT/BAT markers

**Figure 10 f10:**
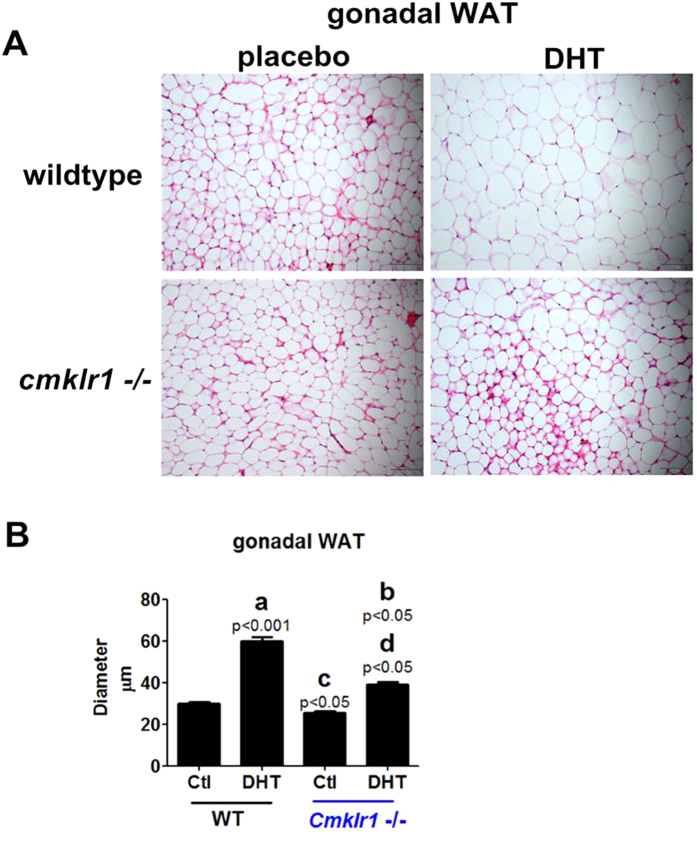
Gonadal white adipose depot features in CTL (placebo) and DHT-treated wild type mice and CMKLR1-deficient mice. Adipose depots were isolated at the end of the 90-d treatment period. (**A**) Hematoxylin and eosin (HE)-stained section of gonadal adipose depots in placebo- and DHT-treated wild type mice and CMKLR1 null mice. Scale bar = 50 μm. (**B**) Adipocyte size measurement in placebo-treated and DHT-treated mice. In each group, the diameters of a total of 450 adipocytes were measured in three randomly selected sections per fat depot per mouse. Data represent mean ± SEM (n = 10 mice per group). Hematoxylin and eosin staining of CTL and DHT-treated gonadal white adipose depots. Increased adiposity upon chronic DHT treatment in wild type mice compared with placebo-treated wild type mice. Decreased adiposity in DHT-treated CMKLR1 null mice compared with DHT-treated wild type mice. (a) *p*, DHT-treated *vs.* control wild type mice; (b) *p*, DHT-treated *vs.* control CMKLR1-null mice; (c) *p*, vehicle-treated CMKLR1 null mice *vs.* vehicle-treated wild type mice; (d) *p*, DHT-treated CMKLR1 null mice *vs.* DHT-treated wild type mice.

Hematoxylin-eosin (HE)-stained sections of gonadal adipose depots isolated at the end of the 90-d treatment period (Fig. 10A) were used to measure the sizes of the depots (Fig. 10B) in placebo- and DHT-treated wild type and CMKLR1 null mice. In each group, the diameters of a total of 450 adipocytes were measured in three randomly selected sections per fat depot per mouse. Our results indicated increased adiposity in DHT-treated wild type mice when compared with placebo-treated mice (Fig. 10A,B; p < 0.001). CMKLR1 KO significantly, although not completely, attenuated the DHT-induced increase in adiposity (Fig. 10A[Fig f10]B; p < 0.05).

**Figure 11:**
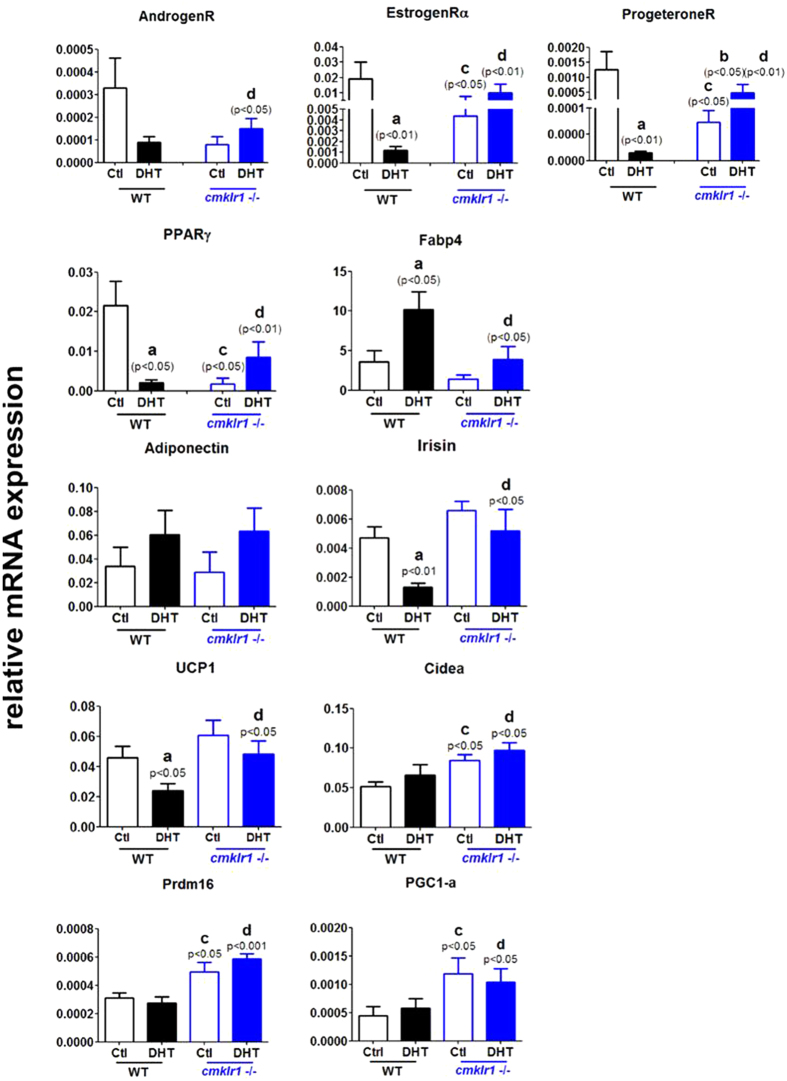
Expression of mRNAs for steroid receptors, white adipose tissue markers, and brown adipose tissue markers in gonadal white adipose tissue of CTL and DHT-treated wild type mice and CMKLR1-deficient mice. Data were analyzed by an unpaired Student’s t test. (a) *p*, DHT-treated *vs.* control wild type mice; (b) *p*, DHT-treated *vs.* control CMKLR1 null mice; (c) *p*, vehicle-treated CMKLR1 null mice *vs.* vehicle-treated wild type mice; (d) *p*, DHT-treated CMKLR1 null mice *vs.* DHT-treated wild type mice.

We next examined the expression of mRNA for steroid receptors and WAT/BAT markers in gonadal WAT (gWAT) (Fig. 11). DHT treatment decreased androgen receptor, estrogen receptor alpha, and progesterone receptor mRNA abundance in wild type gWAT when compared to controls (Fig. 9; p < 0.05, p < 0.01, and p < 0.01, respectively). Although the levels of estrogen receptor alpha and progesterone receptor mRNAs in CMKLR1 null mice were much lower than in wild type mice (Fig. 11; p < 0.05 and p < 0.05, respectively), CMKLR1 KO significantly reversed the reduction in mRNA abundance for all three steroid receptors induced by DHT, as shown in Fig. 11 (p < 0.05, p < 0.01, and p < 0.01, respectively).

Since the balance between WAT and BAT affects the development of follicles, we examined markers for WAT (PPARγ, FABP4, adiponectin, and irisin) and BAT (UCP-1, Cidea, Prdm16, and PGC1-α) in gWAT (Fig. 11). In CMKLR1 wild type mice, DHT increased FABP4 mRNA abundance (Fig. 11; p < 0.05) and decreased PPARγand irisin mRNA levels (Fig. 11; p < 0.05 and p < 0.01, respectively), but had no significant effect on adiponectin mRNA levels. Basal PPARγmRNA levels were much lower in CMKLR1 null gWAT compared with those in wild type gWAT (Fig. 11; p < 0.01), which were significantly increased by DHT treatment (Fig. 11; p < 0.05), although not completely restored to the basal levels of the control wild type group. However, DHT-induced changes in irisin mRNA abundance (Fig. 11; p < 0.01 and p < 0.05, respectively) were abolished in the gWAT of CMKLR1 KO mice.

We also measured mRNA expression levels of brown adipose tissue markers in gWAT; we found that DHT treatment suppressed the mRNA levels of UCP-1 (p < 0.05) but not of Cidea, Pdm16, or PGC1- α in CMKLR1 wild type mice (Fig. 11). The basal and DHT-treated levels of Cidea, Prdm16, and PGC1- α in CMKLR1 KO mice were not significantly different from those in DHT-treated wild type mice (Fig. 11), but the levels in CMKLR1 KO gWAT were significantly higher than those in wild type gWAT, irrespective of DHT treatment (Fig. 11; p < 0.05, p < 0.05, and p < 0.05, respectively).

## Discussion

PCOS is a common endocrine disorder characterized by reproductive, endocrine, and metabolic features, including anovulation, infertility, hyperandrogenism, obesity, hyperinsulinism, and an increased risk of type 2 diabetes and cardiovascular disease^22^. Chemerin, a chemoattractant ligand for CMKLR1^3^,^6^, has been identified as a novel adipokine associated with obesity and metabolic syndrome and has been shown to promote adipogenesis and regulate glucose metabolism^3^,^4^,^7^,^8^. Serum chemerin levels are higher in obese women and women with PCOS. However, the role of chemerin/CMKLR1 in the pathogenesis of PCOS is largely unknown.

It is well known that the reproductive and metabolic phenotypes of human PCOS^19^,^20^ can be mimicked in mice using DHT (90-day treatment), which is characterized by irregular serum estradiol and progesterone levels and abnormalities in cycling in wild type mice. This study presents the first evidence for the preventive/protective effects of CMKLR1 deficiency in the DHT induction mouse model of PCOS. Our data demonstrate that loss of CMKLR1 significantly attenuated the DHT-induced clinical signs of PCOS and alterations in ovarian gene expression in the mouse model. Compared with DHT-challenged wild type mice, DHT-challenged CMKLR1 null mice maintained a relatively normal estrous cycle, higher plasma estrogen and progesterone levels, and multiple corpus lutea, indicative of ovulation. Also, the adipocytes surrounding the ovaries in DHT-challenged CMKLR1-deficient mice were significantly smaller and expressed genes characteristic of brown adipose tissue, suggesting the involvement of the chemerin/CMKLR1 pathway in transition between WAT and BAT. BMP4 inhibition attenuated the induction effects of hCG and DHT on estrogen and progesterone secretion in CMKLR1 KO mice, but not in WT mice, implicating the BMP4 signaling pathway in the CMKLR1-dependent response to DHT. In conclusion, CMKLR1 gene deletion attenuates the damaging effects of chronic DHT treatment on ovarian function in experimental PCOS, and this effect may be mediated by BMP4 signaling. These data provide evidence that CMKLR1 might be a prevention or treatment target for androgen induced-PCOS.

Ovarian steroids play an important regulatory role in ovarian follicular development and atresia^23^. Androgens mediate their actions via the androgen receptor (AR), members of the nuclear receptor superfamily^24^. Women exposed to excess androgen from endogenous (e.g. congenital adrenal hyperplasia)^25^,^26^ or exogenous (testosterone treatment in female-to-male transgenders)^27^ sources display polycystic ovaries, hinting that androgens stimulate follicle development. Further *in vitro* pharmacological studies have shown that testosterone, androstenedione (A4), and dihydrotestosterone (DHT) enhance follicle growth and development^28^,^29^.

Our lab previously reported on the expression of the novel adipokine chemerin and its receptors, CMKLR1 and GPR1, in human testis and characterized its direct biological effects in the mammalian male gonad^30^. Chemerin has also been shown to suppress steroid hormone secretion from human^31^ and rat^13^ granulosa cells, suggesting that chemerin is a regulator of gonadal steroidogenesis. Since androgen contributed to the pathological process of PCOS, and both chemerin and CMKLR1 were elevated in the antral follicles after DHT treatment^12^, we hypothesize that androgen-induced ovarian follicular growth and function are mediated in part by increased expression and action of chemerin. To investigate the role of chemerin in PCOS, we used CMKLR1 KO and wild type mice to establish the DHT-induced PCOS model. We found the following in DHT-treated CMKLR1 KO mice in comparisons with wild type mice: 1) at least one estrous cycle in CMKLR1-deficient mice; 2) more preantral, antral, and preovulatory follicles and fewer atypical follicles in ovaries; 3) high serum estradiol and progesterone levels; 4) less apoptosis in thecal and stromal cells of the antral follicle. These phenotypes suggest that CMKLR1 deficiency protects ovaries from DHT-induced ovary function disorders.

Since CMKLR1 KO affected hormone levels in response to DHT treatment, we examined the key limiting enzymes in steroidogenesis: steroidogenic acute regulatory protein (StAR)^32^,^33^, cytochrome P450scc^34^, and 3β-hydroxysteroid dehydrogenase (3β-HSD)^35^. StAR regulates the transport of cholesterol from the outer to the inner mitochondrial membrane^32^,^33^, P450scc is the key enzyme in the conversion of cholesterol to pregnenolone^34^, and 3β-HSD present in the smooth endoplasmic reticulum converts pregnenolone into progesterone^34^. After DHT treatment, StAR, P450scc, and 3β-HSD were all downregulated in both wild type and CMKLR1 KO mice relative to placebo control mice. However, the reduction in StAR, P450scc, and 3β-HSD induced by DHT was blunted in the ovaries from CMKLR1 null mice, suggesting CMKLR1 deficiency maintained progesterone and subsequent estradiol biosynthesis to some extent. This is consistent with the decrease in progesterone and its subsequent reduction of estradiol, and it may be the reason for the estrous cycle, the increase in preantral, antral, and preovulatory follicles, and the decrease in apoptotic follicles in ovaries. In addition, the ovaries of CMKLR1 null mice express higher levels of insulin receptor (InsulinR) mRNA than do wild type ovaries. Previous studies have shown that insulin induces cell proliferation, steroid production, and StAR, CYP11A1, and CYP17 gene expression, thus promoting steroidogenesis^36^,^37^. In the current study, DHT treatment was found to inhibit progesterone biosynthesis partly by inhibiting insulin receptor mRNA expression in ovaries, whereas CMKLR1 deficiency alleviated the negative effect of DHT by upregulating insulin receptor expression, thus maintaining progesterone production in ovaries.

BMP members are co-expressed in the ovary, and thecal interstitial cells are known to produce at least BMP4, BMP2, BMP7, and GDF10^16^,^38^,^39^. The physiological function of BMP4 is mediated through a type I receptor (BMPRII/ActRIIA) and type II receptors (ALk3 and Alk6)^40^. It has been reported that BMP4 inhibits progesterone synthesis and secretion in ovine ovarian and bovine granulosa cells but that it has no effect on estradiol^41^. Furthermore, BMP4 was found to suppress the expression of StAR at the mRNA and protein levels. DAN, a specific blocker of BMP2 and BMP4, significantly reversed the inhibitory effect of BMP4 on follicle-stimulating hormone-induced progesterone production in cultured rat granulosa cells^41^,^42^,^43^,^44^,^45^. To further determine whether BMP4 is involved in the cellular mechanism of action of chemerin, we examined the effect of CMKLR1 deficiency on the expression of BMPs and their receptors. We found that DHT treatment led to significant upregulation of BMP4, BMPRII, and Alk6 and that DHT-induced upregulation of BMP4 subsequently inhibited progesterone production. In contrast, CMKLR1 null mice expressed more ActRIIA mRNA but exhibited a smaller response to DHT treatment, suggesting that loss of CMKLR1 blocked DHT induction of the BMP4 pathway in ovaries and enabled them to maintain a degree of progesterone synthesis.

To confirm the relationship among CMKLR1, DHT, and DAN, an antral follicle *in vitro* culture assay was performed. hCG treatment upregulated StAR, p450scc, and 3βHSD expression and induced progesterone and estradiol secretion in both wild type and CMKLR1 null follicles. However, there was a different pattern after DHT and/or DAN incubation. The induction effect of hCG on StAR, p450scc, and 3βHSD gene expression and progesterone and estradiol production was partially attenuated by the BMP4-selective blocker DAN in wild type follicles. DHT alone attenuated DAN’s effect on the induction by hCG. In contrast, neither DHT treatment alone nor DAN treatment alone modified the effect of hCG on the expression of steroid synthesis enzyme mRNAs and progesterone/estradiol production in CMKLR1 null follicles. However, in the presence of DHT, DAN dramatically abrogated the stimulatory effects of hCG on StAR, p450scc, and 3βHSD mRNA expression and progesterone/estradiol secretion. These data demonstrate that DHT and DAN act synergistically on CMKLR1 null follicles but not wild type follicles, suggesting that the effect of DHT mediated by the BMP4 pathway differs between wild type and CMKLR1 null follicles. The precise cellular and molecular mechanisms underlying this difference will need further study to be elucidated.

Mounting evidence indicates that adipose tissue dysfunction plays a central role in the metabolic abnormalities observed in PCOS^46^,^47^. Adipose tissue is divided into two types according to their functions: WAT and BAT^48^. WAT and BAT have essentially antagonistic functions: WAT stores excess energy as triglycerides and BAT is specialized for the dissipation of energy through the production of heat. It has been reported that BAT transplantation improves whole-body energy metabolism, glucose homeostasis, and insulin sensitivity^49^,^50^, which may be developed into an effective treatment for age-related obesity^48^,^59^.

Adipocyte-fatty acid-binding protein (FABP4) is an adipokine that regulates systemic insulin sensitivity and lipid and glucose metabolism^51^. In humans serum FABP4 levels correlate significantly with features of PCOS. Uncoupling protein 1 (UCP1) is a defining constituent of brown adipocytes and pivotal for cold- and diet-induced thermogenesis^52^. Peroxisome proliferator-activated receptor gamma coactivator 1-alpha (PGC-1alpha) controls several aspects of mitochondrial biogenesis, especially in brown adipose tissue. Cidea, a close homolog of Fsp27, is expressed at high levels in brown adipose tissue and plays an important role in controlling lipid droplet fusion, lipid storage in brown and white adipose tissue, and the development of obesity_ENREF_50. Prdm16 is the key master gene involved in the switch between myogenic and brown adipogenic lineages^53^. Adiponectin is secreted by fat cells and circulates in the blood. The plasma adiponectin concentration is reduced in obese animals and humans and in patients with type 2 diabetes mellitus and PCOS^54^,^55^,^56^. Irisin is a novel myokine and responsible for exercise-induced browning of white adipose tissue^57^,^58^. Our current data indicate that DHT treatment leads to hypertrophic adipocytes, reduced expression of steroid receptors, PPARγ, irisin, and UCP-1, and increased expression of FABP4 in the gonadal white depots of wild type mice. However, CMKLR1 null mice under chronic DHT conditions have smaller adipocyte size, reduced expression of the white adipose marker FABP4, and increased expression of mRNAS for steroid receptors, PPARγ, irisin, and brown adipose tissue markers (UCP-1 and Prdm16) compared with DHT-treated wild type mice, suggesting that gonadal white adipocytes are more prone to browning in DHT-treated CMKLR1-deficient mice. Such brown-like adipocytes may secrete some substances, such as irisin, but not adiponectin, so that they are helpful for follicle development, steroid biosynthesis, and ovarian function. However, the detailed mechanism needs to be further elucidated.

**Figure 12:**
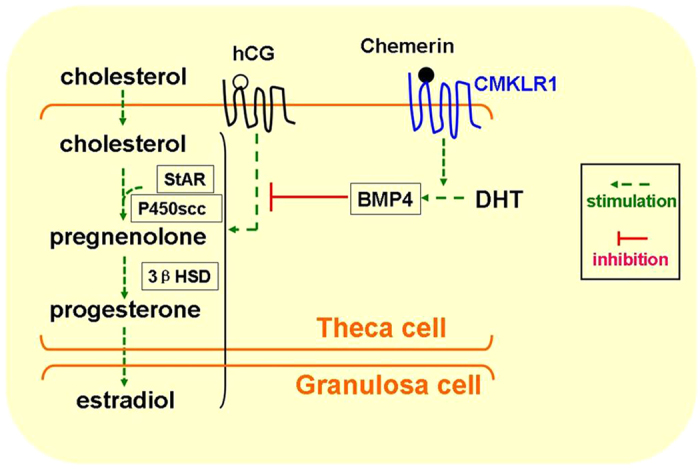
Modulation of progesterone and estradiol biosynthesis in follicles with DHT treatment. The process of progesterone production requires the following enzymes: cholesterol side-chain cleavage cytochrome P450 (CYP11A1, P450scc), 3-βhydroxysteroid dehydrogenase (3βHSD), and steroidogenic acute regulatory protein (STAR). Thecal cells from rodents produce progesterone/testosterone as precursors for estradiol production in neighboring granulosa cells. Expression levels of genes encoding steroidogenic enzymes are regulated by many different factors within the antral follicle, including hCG, the stimulatory factors are shown in Green arrow. DHT treatment inhibits hCG induction through BMP4 pathway in red T shapes, while Chemerin stimulates the effect of DHT through its receptor, CMKLR1. hCG, Human chorionic gonadotropin. DHT, dihydrotestosterone. BMP-4, bone morphogenetic protein 4.

In conclusion, follicular development is tightly regulated by gonadotropins, cytokines, and growth factors via crosstalk between granulosa cells, thecal cells, the oocyte, and even adipose tissues. In this study, we are the first to demonstrate a role for the chemerin/CMKLR1 pathway in follicle function and steroidogenesis. More important, we found that CMKLR1 deficiency partially ameliorated the negative effect of DHT on progesterone secretion, cycling, and anovulation in mice treated chronically with DHT (Fig. 12). The reason that CMKLR1 deficiency does not completely prevent DHT’s effect may be due to the complicated physiology and pathological roles and needs of chemerin and its receptors, which is beyond the scope of this study and needs to be further elucidated in future studies. Taken together, CMKLR1 gene deletion protects against the damage caused by chronic DHT treatment on ovarian function, might partly through BMP4 signaling. This finding indicates that androgen-induced polycystic ovary syndrome might be prevented through therapeutic targeting of CMKLR1.

## Additional Information

**How to cite this article**: Tang, M. *et al.* CMKLR1 deficiency maintains ovarian steroid production in mice treated chronically with dihydrotestosterone. *Sci. Rep.* **6**, 21328; doi: 10.1038/srep21328 (2016).

## Figures and Tables

**Table 1:** primers list.

Name	Sequence (5′-3′)
mouse Fabp4-Fw	AAGGTGAAGAGCATCATAACCCT
mouse Fabp4-Rev	TCACGCCTTTCATAACACATTCC
mouse Adiponectin-Fw	TGTTCCTCTTAATCCTGCCCA
mouse Adiponectin-Rev	CCAACCTGCACAAGTTCCCTT
Mouse Cidea-Fw	TGCTCTTCTGTATCGCCCAGT
Mouse Cidea-Rev	GCCGTGTTAAGGAATCTGCTG
Mouse PGC-1α-Fw	AGCCGTGACCACTGACAACGAG
Mouse PGC-1α-Rev	GCTGCATGGTTCTGAGTGCTAAG
Mouse PRDM16-Fw	CAGCACGGTGAAGCCATTC
Mouse PRDM16-Rev	GCGTGCATCCGCTTGTG
Mouse UCP1-Fw	ACTGCCACACCTCCAGTCATT
Mouse UCP1-Rev	CTTTGCCTCACTCAGGATTGG
mouse Alk3 -Fw	CCTGTTGTTATAGGTCCGTTCTT
mouse Alk3-Rev	AGCTGGAGAAGATGATCATAGCA
mouse Alk6 -Fw	GGAAGACTCAGTCAACAATATCTGC
mouse Alk6-Rev	CTAGTCCTAGACATCCAGAGGTGAC
mouse ActRIIA -Fw	GTTGAACCTTGCTATGGTGATAA
mouse ActRIIA-Rev	AATCAGTCCTGTCATAGCAGTTG
mouse BMPRII-Fw	AGCTGACAGAAGAAGACTTGGAG
mouse BMPRII-Rev	CAAGCTAGAACTGGTACTGCTCA
mouse bmp2 -Fw	ACTTTTCTCGTTTGTGGAGC
mouse bmp2-Rev	GAACCCAGGTGTCTCCAAGA
mouse bmp4 -Fw	GAGGAGGAGGAAGAGCAGAG
mouse bmp4-Rev	TGGGATGTTCTCCAGATGTT
mouse bmp6 -Fw	AACCTTTCTTATCAGCATTTACCA
mouse bmp6 -Rev	GTGTCCAACAAAAATAGGTCAGAG
mouse bmp7 -Fw	GGGCTTACAGCTCTCTGTGG
mouse bmp7 -Rev	TGAAGGGTTGCTTGTTCTGG
Mouse- StAR-fw	CCGGGTGGATGGGTCAA
Mouse- StAR-Rev	CACCTCTCCCTGCTGGATGTA
Mouse-P450scc-Fw	CCATCAGATGCAGAGTTTCCAA
Mouse-P450scc-Rev	TGAGAAGAGTATCGACGCATCCT
Mouse-3β-HSD-Fw	GGAGGCCTGTGTTCAAGCAA
Mouse-3β-HSD-Rev	GGCCCTGCAACATCAACTG
mouse estrogen receptor alpha-Fw	CCTCCCGCCTTCTACAGGT
mouse estrogen receptor alpha-Rev	CACACGGCACAGTAGCGAG
mouse estrogen receptor beta-Fw	CTGTGATGAACTACAGTGTTCCC
mouse estrogen receptor beta-Rev	CACATTTGGGCTTGCAGTCTG
mouse androgen receptor-Fw	CTGGGAAGGGTCTACCCAC
mouse androgen receptor-Rev	GGTGCTATGTTAGCGGCCTC
mouse progesterone receptor-Fw	CTCCGGGlACCGAACAGAGT
mouse progesterone receptor-Re	ACAACAACCCTTTGGTAGCAG
mouse beta-Actin-Fw	GTATCCATGAAATAAGTGGTTACAGG
mouse beta-Actin-Re	GCAGTACATAATTTACACAGAAGCAAT
